# Coexisting Mantle Cell Lymphoma and Prostate Adenocarcinoma

**DOI:** 10.1155/2014/247286

**Published:** 2014-10-16

**Authors:** Ashish B. Rajput, Bruce Burns, Ronald Gerridzen, Richard van der Jagt

**Affiliations:** ^1^Division of Hematopathology, The Ottawa Hospital, General Campus, 501 Smyth Road, Ottawa, ON, Canada K1H 8L6; ^2^Eastern Ontario Regional Laboratory Association and The Ottawa Hospital, General Campus, 501 Smyth Road, Ottawa, ON, Canada K1H 8L6; ^3^Department of Pathology and Laboratory Medicine, Division of Hematopathology and Transfusion Medicine, The Ottawa Hospital, General Campus, 501 Smyth Road, Ottawa, ON, Canada K1H 8L6; ^4^Division of Anatomical Pathology, The Ottawa Hospital, General Campus, 501 Smyth Road, Ottawa, ON, Canada K1H 8L6; ^5^Division of Urology, The Ottawa Hospital, Civic Campus, 1053 Carling Avenue, Ottawa, ON, Canada K1Y 4E9; ^6^Division of Hematology, The Ottawa Hospital, General Campus, 501 Smyth Road, Ottawa, ON, Canada K1H 8L6

## Abstract

Prostatic mantle cell lymphoma (MCL) is a very rare entity with only 5 reported cases in the literature. We report a case of coexisting MCL and prostate adenocarcinoma (PCa) in an elderly male and review the morphologic features of classic and rare prostatic MCL subtypes. Careful morphologic evaluation and immunohistochemical findings of positive CD5, CD20, and cyclin D1 and negative CD23 and CD3 can guide us to the diagnosis of MCL. Given the fact that transurethral resection of prostate is done quite routinely, this paper draws attention to the manner in which long standing bladder outlet obstruction and postbiopsy prostate specimens with dense lymphoid infiltration can masquerade as lymphoma. It highlights the importance of exercising care while reviewing prostate specimens with evidence of chronic prostatitis so as not to miss this rare neoplasm.

## 1. Introduction

Mantle cell lymphoma (MCL) is a lymphoproliferative disorder accounting for about 3–10% of all non-Hodgkin lymphomas [1]. MCL involving the prostate gland is very rare with only five reported cases.

We report incidental finding of MCL in a Gleason grade 6 (3 + 3) prostate adenocarcinoma (PCa). The patient had initially presented with the signs and symptoms of bladder outlet obstruction 10 years ago and underwent transurethral resection of prostate (TURP), and a histologic diagnosis of benign prostate hyperplasia (BPH) was made at that time. He presented this time with similar symptoms and markedly elevated prostate specific antigen (PSA) levels. A core biopsy was obtained to confirm PCa. Besides the obvious PCa, there was evidence of chronic prostatitis due to previous biopsy and recurrent bladder outlet obstruction. Careful morphologic examination of core tissue biopsy coupled with immunohistochemistry (IHC) led to the diagnosis of MCL, which was later confirmed by a bone marrow aspirate, biopsy, and flow cytometry. This case adds to the growing knowledge of presentation of this rare entity and highlights the importance of careful microscopic examination of lymphocytic infiltration and use of IHC to diagnose MCL, especially in the setting of chronic prostatitis.

## 2. Case Report

We present a case report of coexisting MCL and PCa in a 74-year-old male patient. The patient first presented in 2003 at the age of 63 years with increased frequency and nocturia. He underwent TURP and 26 grams of prostate curetting examined for histopathologic abnormality. The stained slides showed nodular proliferations of glandular and fibromuscular tissue admixed with chronic inflammatory infiltrate and a diagnosis of BPH was made at that time.

He presented 10 years later in July 2013 with elevated PSA level of 17.16 *μ*g/L (normal <3.0 *μ*g/L) and an enlarged prostate on digital rectal examination. An ultrasound revealed a prostate of 116 mL with no focal lesion and splenomegaly of 14.8 cm. A staging CT of the thorax, abdomen, and pelvis confirmed splenomegaly but showed no evidence of intrathoracic or intra-abdominal disease or involvement of LN. A CT head with contrast was normal. The patient also had systemic lupus erythematosus involving both the lungs and rib cage causing significant arthralgias, pleuritic chest pain, and fatigue. However, he had no weight loss, night sweats, or fevers and continued to remain active. His only treatment included Plaquenil and Finasteride. The patient was a retired heavy equipment mechanical operator, nonsmoker, and nondrinker. His family history revealed no history of autoimmune disorders, cancers, or lupus.

The blood work showed white blood cell count of 4.2 × 10^9^/L (normal: 3.5–10.5 10^9^/L), consisting of 3.0 × 10^9^/L (normal: 2.0–7.5 × 10^9^/L) neutrophils, 0.6 × 10^9^/L (normal: 0.8–3.5 × 10^9^/L) lymphocytes, and 0.9 × 10^9^/L (normal: 0.1–1.0 × 10^9^/L) monocytes. The hemoglobin was 8 g/dL (normal: 12.5–17.0 g/dL), hematocrit was 0.241 (normal: 0.38–0.5), and platelet count was 49 × 10^9^/L (normal: 130–380 × 10^9^/L). The serum lactate dehydrogenase was 169 U/L (normal: 100–205 U/L). Blood coagulation profile, serum electrolytes, and liver and renal function tests were within normal limits. The patient's Eastern Cooperative Oncology Group (ECOG) performance status was 0. Based on these parameters, the patient's mantle cell lymphoma international prognostic index score was calculated as 1 (low risk 0–3).

**Figure 1:**
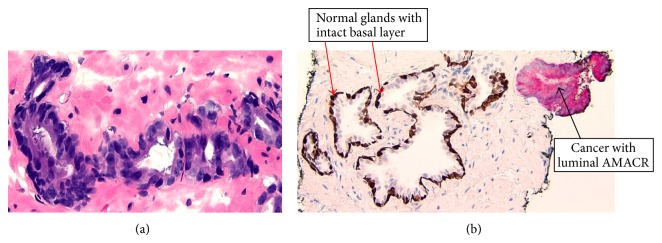
(a) Prostate adenocarcinoma Gleason grade 6 (3 + 3), 20x hematoxylin and eosin. (b) Normal glands have intact basal layer stained dark brown with CK 5/6. Cancer tissue lacks basal layer and luminal glands show finely granular pink cytoplasmic stain, 20x PIN4 cocktail.

In light of increased PSA levels and prostatic enlargement, core needle biopsy of the prostate was performed in November 2013. It showed adenocarcinoma (acinar, not otherwise specified) involving left base of the gland (Figure 1(a)) with a Gleason grade 6 (3 + 3). There was no evidence of seminal vesicle, lymphatic, or perineural invasion. Immunohistochemical staining with triple stain PIN4 cocktail comprising of* alpha*-methylacyl-CoA racemase (AMACR), p63, and CK 5/6 confirmed areas of PCa and delineated the normal prostate glands. Normal glands with intact basal layer showed dark brown nuclear stain and absent AMACR staining. The PCa areas showed cytoplasmic red finely granular staining pattern and absence of basal layer (Figure 1(b)).

**Figure 2:**
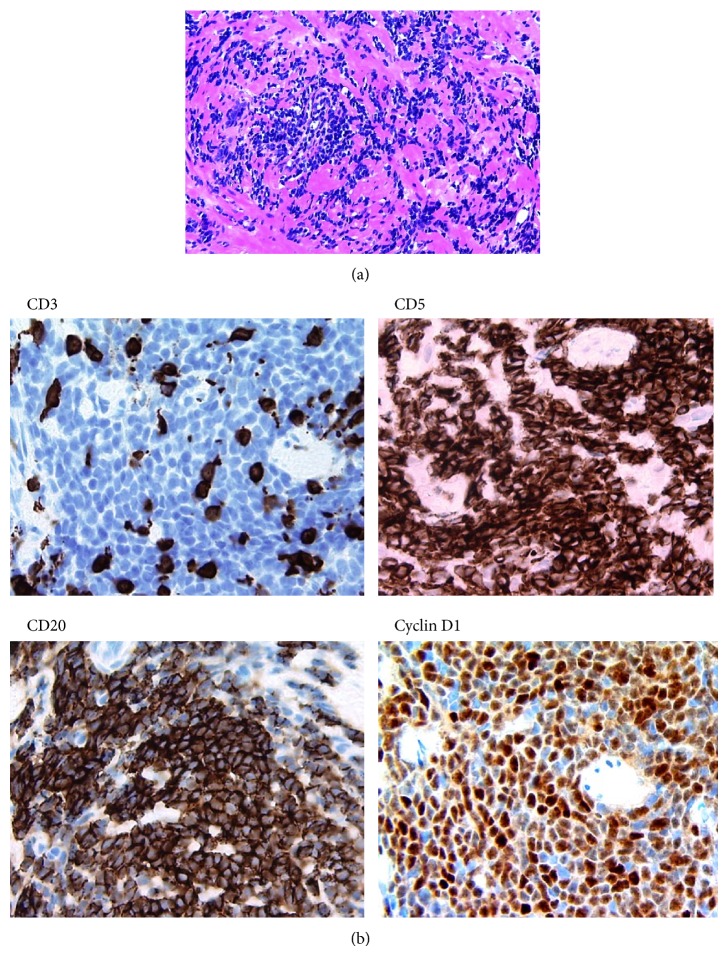
(a) Prostate mantle cell lymphoma, 20x hematoxylin and eosin. (b) Immunohistochemical stains for mantle cell lymphoma, 40x CD3, CD5, CD20, and cyclin D1.

Incidentally, it was found that several cores from right base, right apex, and left base showed infiltrate of small lymphocytes in the stroma of the prostate. The cells were monomorphic with round nuclei, compact chromatin, inconspicuous nucleoli, and no evidence of cytological atypia or mitotic activity (Figure 2(a)). Immunohistochemical analysis revealed strong positivity for CD5 and CD20. Ki67 was less than 10%. Cyclin D1 positivity was noted in 80% of the lymphoid cells. The cells were negative for CD3, CD23, thyroid transcription factor-1, and PSA (Figure 2(b)). These findings lead to the diagnosis of MCL of the prostate.

In January 2014, a bone marrow aspirate from right posterior iliac crest showed mild lymphocytosis, increased marrow cellularity, 1 : 1 GE ratio, and 60% small lymphocytes with irregular nuclear borders. Blasts accounted for 2% of the population. Flow cytometry revealed 42% of the events with kappa light chain restriction, and 50% of these clonal B cells were CD19/CD5 positive. CD23 and CD10 were negative. The bone marrow biopsy showed neoplastic lymphoid infiltrate in 50% of the biopsy specimen with interstitial and paratrabecular distribution. IHC showed CD5, CD20, and cyclin D1 positivity. The bone marrow aspirate and biopsy report were diagnostic of MCL.

Our patient had involvement of the bone marrow and prostate and no systemic symptoms of fever, night sweats, or 10% weight loss in 6 months preceding admission and was thus classified as Ann Arbor stage IVA MCL. He is receiving bendamustine-rituximab chemotherapy since January 2014.

## 3. Discussion

MCL lymphomas of the prostate are rare events with only five cases reported in the literature [2–6]. MCL is a B cell neoplasm arising from a subset of naive pregerminal centre cells of primary follicles or in the mantle region of secondary follicles. The incidence of MCL is 5 cases per 100,000 individuals per year, with male to female ratio of approximately 2.4 : 1 [7]. The median age of presentation is around 60 years (range: 35–85 years). The most common site of involvement is the lymph node (LN). The median survival is 3–5 years, the 10-year survival rate is only 5–10%, and most cases are not curable [8].

Histopathology of a classic MCL shows expansion of the mantle zone surrounding the LN germinal centre by monomorphic lymphocytes arranged in patterns ranging from vaguely nodular, diffuse follicular, and mantle zone to diffuse. In the vast majority of cases, there are small to medium sized lymphocytes with irregular nuclear contours, mimicking centrocytes of a germinal centre. The nuclear chromatin is dispersed and the nucleolus is often inconspicuous.

Morphologic variants include the aggressive blastoid and pleomorphic types. The “blastoid” variant shows medium to large cells that closely resemble lymphoblasts with dispersed chromatin and a high mitotic rate of 20–30/10 hpf. A case report of a patient with lung and prostate cancer who developed blastic variant MCL, while being treated with imatinib mesylate for chronic myeloid leukemia, is described in the literature [9]. The “pleomorphic” variant shows larger cells with oval or irregular nuclear contour, pale cytoplasm, and some cells with prominent nucleoli.

Other variants include the “small cell” and “marginal zone-like” type. The “small cell” type has small round lymphocytes with clumped chromatin mimicking a small lymphocytic lymphoma. The “marginal zone-like” variant has prominent foci of cells with abundant pale cytoplasm resembling marginal zone or monocytoid B cells seen in marginal zone lymphoma [10].

IHC shows intense surface IgM/IgD (lambda restriction more common than kappa). Cells are usually positive for CD5, CD19, CD20, and CD22 which is consistent with their small B lymphocyte origin. They often lack CD23, BCL6, and CD10. Almost all cases show BCL2 positivity and express cyclin D1 which is involved in G1 to S phase cell cycle progression through activation of cyclin-dependent kinases.

The cytogenetic abnormality underlying MCL involves t (11; 14) (q13; q32) translocation between immunoglobulin heavy-chain* IGH* gene on chromosome 14 and the* BCL1* locus on chromosome 11. This results in deregulated overexpression of* CCND1* and, in some cases, increased half-life of cyclin D1. This results in loss of cell cycle suppressive effects of retinoblastoma protein RB1 and p27kip1, resulting in MCL development [10].

Thus the diagnosis of MCL relies on combination of morphologic and immunohistochemical features. Lymphoid follicles may not be readily appreciated and can be misdiagnosed as chronic prostatitis. The patient underwent TURP 10 years prior to a final pathologic diagnosis of BPH. At the time of this presentation, he again had symptoms of bladder outlet obstruction and very high PSA levels which prompted the treating physician to do a diagnostic core prostate biopsy. Besides the obvious PCa, we discovered MCL on routine hematoxylin and eosin staining and confirmed it with IHC.

It is known that chronic prostatitis occurring as complication of bladder outlet obstruction or after an old prostate biopsy is a common finding. Extensive chronic inflammation in such cases may mimic prostatic lymphoma [11]. In doubtful cases, IHC special stains help delineate the diagnosis. Our patient was incidentally diagnosed when we carefully examined the lymphocytic infiltration in an otherwise straightforward PCa specimen. IHC staining with CD5, CD20, and cyclin D confirmed the diagnosis of MCL. Also negative staining with CD23 ruled out the diagnosis of chronic lymphocytic lymphoma.

## 4. Conclusion

Given the fact that TURP is done quite routinely and MCL of prostate is such a rare entity, this report emphasizes the importance of carefully inspecting for evidence of chronic prostatitis. In the absence of systemic symptoms and normal peripheral blood counts, postbiopsy prostate specimens with dense lymphoid infiltration may masquerade as lymphoma. A high degree of suspicion coupled with morphologic and IHC analysis showing positive CD5, CD20, and cyclin D1 and negative CD23 and CD3 can guide us to the diagnosis of MCL.
